# Challenges and advances of implementing affirmative action policies within graduate nursing programs

**DOI:** 10.3389/fpubh.2025.1714039

**Published:** 2026-01-09

**Authors:** Ygor de Oliveira Navarro da Conceição, Bruno Pereira da Silva, Frantz Rousseau Deus, Debora Souza Santos, Maria Giovana Borges Saidel

**Affiliations:** 1 School of Nursing, State University of Campinas (UNICAMP), Campinas, SP, Brazil; 2 Multidisciplinary Center, Federal University of Acre (UFAC), Cruzeiro do Sul, AC, Brazil; 3 Graduate Program in Education and Health in Childhood and Adolescence (PPGESIA), Federal University of São Paulo (Unifesp), Guarulhos, SP, Brazil

**Keywords:** affirmative action, Brazil, graduate education, implementation, nursing, racial equity

## Abstract

**Background:**

Affirmative action (AA) policies are increasingly used in Brazilian graduate programs to address racial inequities. However, implementation is often fraught with institutional tensions, conceptual ambiguities, and uneven adherence. This study investigates the challenges and progress in implementing AA within a Graduate Nursing Program in Brazil.

**Methods:**

We conducted a qualitative study with seven academic managers who held positions (coordinators, associate coordinators, and committee members) within the program between 2015 and 2024. Recruitment was guided by purposive sampling, complemented by a snowball strategy. Data were collected through in-depth interviews and analyzed using inductive thematic analysis.

**Results:**

Four interconnected thematic axes emerged: (1) diverse interpretations and conceptual dilemmas surrounding AA; (2) institutional facilitators and barriers, including concerns about CAPES evaluation indicators, a lack of binding regulations, and insufficient legal support; (3) the process leading to AA adoption, which was shaped by internal disagreements and external initiatives, such as the Uhayele project; and (4) the limits of access-centered policies, specifically the absence of structured student retention strategies. Although managers recognized AA as crucial for promoting racial equity, implementation was constrained by normative gaps, meritocratic narratives, and the homogeneity of leadership.

**Conclusion:**

AA effectiveness in graduate education requires embedding it within broader institutional projects supported by clear guidelines, legal backing, and robust retention strategies. Universities must strengthen mechanisms like scholarships and psychosocial support and establish permanent inclusion committees for consistency. The study provides original insights into AA implementation dynamics in the Global South, contributing to global debates on equity in health.

## Introduction

Affirmative action (AA) policies have become a central mechanism for addressing historical and structural racial inequities within the Brazilian higher education system. Since the approval of Law 12.711/2012, public universities have progressively adopted quota-based systems to expand access for Black, Indigenous, and socioeconomically disadvantaged students. These policies have extended beyond undergraduate education, increasingly driving discussions about diversity, equity, and inclusion in postgraduate education (1). However, the incorporation of AA in graduate programs has proceeded unevenly across institutions, reflecting a complex interplay of political, administrative, and conceptual challenges (2, 3).

The successful implementation of AA in postgraduate programs requires not only regulatory adjustments but also significant shifts in institutional culture, governance, and pedagogical practices. Academic managers play a pivotal role in mediating these transformations, as they are tasked with interpreting national legislation, negotiating local norms, and integrating AA into selection processes, academic routines, and, crucially, retention strategies (3, 35). Consequently, their perceptions and decision-making processes offer critical insights into how AA is understood, contested, and operationalized within academic institutions.

At the University of Campinas (Unicamp), the adoption of AA in postgraduate programs advanced through decentralized initiatives, ultimately leading to the institutionalization of racial quotas via Deliberação CONSU-A-008/2023. Within this context, the School of Nursing (FEnf) implemented the Uhayele Project in 2023. Funded by the Graduate Program Development Program (PDPG)—Alterity in Postgraduate Education, this project aimed to promote racial equity, strengthen institutional capacity, and support the development of AA policies within the Graduate Nursing Program (PPG-Enf). The project created a timely opportunity to examine how managers conceptualized affirmative action, identified institutional barriers, and navigated the tensions between equity goals and established academic performance indicators.

Despite these advancements, the adoption of AA in postgraduate nursing programs remains marked by conceptual ambiguities, uneven institutional readiness, and limited mechanisms for ensuring the retention of Black students (3, 36). Prior studies emphasize that the success of AA depends not only on access policies but also on robust institutional support structures that promote academic success, psychosocial well-being, and equitable learning conditions (4, 5). However, there is a distinct lack of knowledge regarding how managers in postgraduate nursing programs interpret the core goals of AA, negotiate internal and external institutional pressures, and construct local policies in practice.

This study addresses this gap by investigating the challenges and progress in implementing AA within a Graduate Nursing Program in Brazil. Through qualitative analysis, the study explores how managers interpret AA within a context characterized by racial inequities, administrative decentralization, and institutional change. By illuminating the conceptual tensions, ethical dilemmas, and organizational challenges that shape AA implementation, this research contributes to a broader understanding of equity-oriented policies in graduate nursing education and offers insights relevant to other programs engaged in similar institutional transformation processes.

## Methods

### Study design

This research employed a qualitative, exploratory, and descriptive study design, grounded in a constructivist perspective. This approach was selected to facilitate the in-depth understanding of complex institutional processes and the meanings attributed by academic managers to their practices, recognizing that reality is socially constructed and emerges from the interaction between researchers and participants (6). The study adheres to the Consolidated Criteria for Reporting Qualitative Research (COREQ) (7) to ensure rigor, transparency, and methodological consistency.

### Setting and context: the Uhayele project

The research was conducted within the PPG-Enf at Unicamp, an academic unit established in 1999 (master’s level) and expanded in 2008 (Doctoral level), located in Campinas, São Paulo, Brazil.

The implementation of AA in the PPG-Enf was integral to the broader Uhayele Project. This institutional initiative was designed to promote racial equity in postgraduate education at the School of Nursing (FEnf/Unicamp), with the overarching goal of expanding access, inclusion, and retention of self-declared Black students. The project combined research, management, and pedagogical strategies.

Its specific objectives included: (1) mapping barriers and facilitators to the adoption of AA within the program; (2) supporting the design and implementation of reserved positions for Black applicants in the selection process; and (3) developing initial strategies to monitor access and retention of students admitted via AA policies.

Key activities encompassed diagnostic meetings with academic managers, analysis of institutional regulations, participatory discussions within the Graduate Program Committee, and the revision of admissions calls to incorporate racial quotas into the regular selection process. The Uhayele Project received funding from the Brazilian Coordination for the Improvement of Higher Education Personnel (CAPES) through the Graduate Program Development Program (PDPG) – Alterity in Postgraduate Education (Call no. 37/2022), a program specifically supporting equity, diversity, and inclusion initiatives in postgraduate education.

### Participants and sampling

Participants were academic managers and faculty members who served on the Graduate Program Committee (CPG) of the PPG-Enf from 2015 to 2024. A total of twelve managers met the inclusion criteria and were invited via institutional email. Of these, seven agreed to participate, four declined due to scheduling constraints, and one did not respond.

Sampling was guided by a purposive strategy, consistent with qualitative health research (3), focusing on individuals directly involved in decision-making processes related to AA. This initial recruitment was complemented by a snowball procedure, where participants were asked to indicate additional managers who played relevant roles in AA discussions. This process generated three new referrals, all of whom overlapped with the initial purposive invitations, reinforcing the consistency between formal managerial roles and the program’s informal decision-making networks.

The sample size was determined by theoretical saturation, as proposed by Guest et al. (8). Saturation was achieved after the seventh interview, when no new codes, perspectives, contradictions, or operational challenges emerged from the data. For instance, the final two interviews consistently reiterated established themes—such as tensions between ethical support for AA and concerns about CAPES performance indicators—without introducing new conceptual elements, confirming the dataset’s richness for addressing the study’s objectives.

### Data collection

Data were collected between July 2023 and March 2024 through semi-structured interviews conducted in a virtual environment. This technique enabled an in-depth exploration of participants’ perceptions while maintaining a minimum level of standardization (9). The interview guide was collaboratively developed by the research team and refined after two pilot interviews to optimize clarity and relevance. The guide included three main sections: (1) participant characterization, (2) sociodemographic information, and (3) perceptions and experiences related to AA policies. All interviews were audio-recorded, fully transcribed, and anonymized.

### Data analysis

Thematic Analysis was performed following Braun and Clarke’s (10) six-step framework, supported by NVivo 1.3 Release software. The process involved: (1) data familiarization; (2) initial coding; (3) generation of themes; (4) review of themes; (5) definition and naming of themes; and (6) production of the analytical narrative. Codes were derived both inductively from participants’ accounts (*in vivo*) and deductively from conceptual categories established in the literature. To ensure rigor, coding was discussed among researchers, with discrepancies resolved by consensus. Triangulation of theoretical perspectives and peer validation strengthened internal consistency. Furthermore, reflective journals were maintained to monitor researcher influence on the interpretative process, as proposed by Lincoln and Guba (11).

### Methodological rigor and ethical considerations

Credibility was ensured through prior validation of the interview guide, achievement of theoretical saturation, peer debriefing, and team reflexivity. Transferability was addressed through a detailed description of the institutional context and participant profiles. Dependability and confirmability were maintained via systematic documentation of analytic decisions and the use of specialized software for data management (11).

The study was approved by the Research Ethics Committee of Unicamp (approval no. 6.931.510/2023), adhering to Resolution No. 466/2012 of the Brazilian National Health Council and the Declaration of Helsinki. All participants were informed about the study’s objectives and procedures and provided electronic informed consent prior to participation.

## Results

### Sample characteristics

A total of seven academic managers participated in the study, comprising three graduate program coordinators, two associate coordinators, and two members of the CPG. All participants self-identified as White women, aged between 30 and 65 years, with management experience ranging from 1 to 4 years. Six participants completed their undergraduate degrees in public institutions, and one in a private institution.

### Overview of themes

The thematic analysis identified four interconnected themes that illustrate how managers conceptualized AA, navigated institutional barriers, and negotiated the adoption of these policies within the Graduate Nursing Program. These themes, presented below with their respective subthemes and illustrative excerpts, reveal a complex landscape shaped by conceptual ambiguity and institutional constraints.

### Theme 1—Divergent and fragmented understandings of affirmative action

Managers demonstrated heterogeneous and sometimes conflicting conceptualizations of AA, spanning from deep ethical commitments to racial justice to more abstract or purely administrative interpretations. These divergent meanings fundamentally shaped each manager’s stance regarding the adoption of AA within the program.

### Subtheme 1.1—affirmative action as ethical and historical reparation

Some participants recognized AA as a crucial mechanism to redress historical racial inequalities and expand access for structurally marginalized groups:

*“…these policies were designed to guarantee access… especially for groups that historically experienced social disadvantage, such as Black and Indigenous peoples.”* (E2).

This view aligns with broader national debates on racial justice and reflects a critical understanding of Brazil’s structural racism.

### Subtheme 1.2—affirmative action as administrative equalization

Other managers adopted a more bureaucratic interpretation, framing AA as a general measure to equalize opportunities, often without explicit reference to race:

*“I understand them as measures so people can have the same access regardless of race or other social situations.”* (E2)

This level of abstraction risked diluting the essential racial dimension of AA’s reparative mandate.

### Subtheme 1.3—tensions between race and socioeconomic disadvantage

A recurring dilemma involved the conflation of racial inequities with socioeconomic hardship, notably the argument that AA should encompass socioeconomically disadvantaged White students:

*“Poor white students also struggle… how do we maintain fairness?”* (E4)

This tension highlights persistent misconceptions regarding the specific, reparative intent of racial quotas.

### Theme 2—Institutional ambiguities and uneven implementation across the university

Managers reported that while the central administration publicly endorsed AA, the absence of binding or enforceable university-wide regulations resulted in a highly uneven implementation across different academic units.

### Subtheme 2.1—central support without enforceability

Participants perceived a lack of congruence between the institutional discourse and concrete, consistent action, as implementation responsibility was largely delegated to individual programs:

*“The Pro-Rectorate understood its importance… but each coordinator was expected to implement it at their own pace.”* (E1)

This delegation led to variable timelines and decentralized decision-making processes.

### Subtheme 2.2—exposure to more advanced units as a source of pressure

Discussions and meetings with units further along in AA adoption generated both motivation and institutional discomfort:

*“Some schools were more advanced and shared their experiences… but for us it felt like we were behind.”* (E1)

These comparisons underscored the existing inequalities in institutional readiness across different graduate programs.

### Subtheme 2.3—retention as an overlooked institutional responsibility

Managers emphasized significant gaps in institutional support for retention policies:

*“There was talk about stipends, but nothing concrete about housing, support, or other measures.”* (E3)

This finding reflects a recurrent mismatch between policies focusing on access and those designed for permanence and academic success.

### Theme 3—Internal dynamics and delays in adoption within the School of Nursing

Within the Graduate Nursing Program, the implementation of AA unfolded slowly, heavily shaped by internal administrative priorities and operational uncertainties.

### Subtheme 3.1—AA as a non-priority agenda item

Participants recalled that AA was discussed only sporadically, frequently overshadowed by other pressing administrative demands:

*“I don’t remember it being a formal agenda item… other issues were prioritized.”* (E1)

This deprioritization significantly contributed to delays in decision-making and policy advancement.

### Subtheme 3.2—operational uncertainties overshadowing conceptual agreement

Even where there was ethical agreement with the goals of AA, the lack of clear procedural guidance produced hesitation and institutional inertia:

*“We didn’t have a legal team to consult… only other faculty.”* (E1)

These operational uncertainties created a major barrier to effective implementation.

### Subtheme 3.3—narrow conceptualization of retention

Retention support was frequently and narrowly equated solely with financial stipends (e.g., the PED program):

*“Here, we basically think about the PED stipend… trying to prioritize that.”* (E1)

This limited view overlooks the broader necessity for comprehensive academic, psychosocial, and structural support crucial for student success.

### Theme 4—Ethical dilemmas, misconceptions, and conceptual limits in AA construction

The process of constructing AA policies brought to the surface deep-seated ethical tensions related to race, the concept of merit, and perceived notions of fairness.

### Subtheme 4.1—confusion between racial reparations and socioeconomic vulnerability

Although often framed as a concern for general fairness, the persistent argument for including low-income White individuals reflects fundamental conceptual misunderstandings of AA’s core purpose:

*“Poor white students also struggle… how do we maintain fairness?”* (E4)

This reframing effectively obscures the racialized nature of structural inequality that AA policies are designed to address.

### Subtheme 4.2—fear of institutional repercussions (CAPES indicators)

Concerns regarding the potential negative impact of AA on national performance metrics—specifically those set by CAPES—significantly influenced decision-making:

*“We worried about the impact on indicators… it was a real concern.”* (E2)

These structural pressures related to institutional evaluation demonstrably shaped managers’ hesitation regarding AA adoption.

### Subtheme 4.3—resistance framed as caution or neutrality

Some resistance to AA appeared in indirect forms, often cloaked in the language of procedural doubt or necessity for caution:

*“We needed to be careful… to avoid harming anyone.”* (E4)

This discursive pattern aligns with documented forms of covert institutional resistance described in the literature.

Table 1 summarizes the four analytic themes identified through thematic analysis, along with their respective subthemes and selected excerpts that illustrate managers’ perceptions, dilemmas, and interpretations regarding the implementation of affirmative action policies in the Graduate Nursing Program.

**Table 1:** Themes, subthemes, and illustrative excerpts related to the implementation of affirmative action policies.

Theme	Subtheme	Illustrative excerpts
1. Divergent and fragmented understandings of affirmative action	1.1. AA as ethical and historical reparation	*“These policies were designed to guarantee access… especially for groups historically experiencing disadvantage.”* (E2)
	1.2. AA as administrative equalization	*“Measures so people can have the same access regardless of race or social situation.”* (E2)
	1.3. Tensions between race and socioeconomic vulnerability	*“Poor white students also struggle… how do we maintain fairness?”* (E4)
2. Institutional ambiguities and uneven implementation across the university	2.1. Support without enforceability	*“The Pro-Rectorate understood its importance… but each coordinator was expected to implement it at their own pace.”* (E1)
	2.2. Exposure to advanced units causing pressure	*“Some schools were more advanced… for us it felt like we were behind.”* (E1)
	2.3. Retention as an overlooked responsibility	*“There was talk about stipends, but nothing concrete about housing or other support.”* (E3)
3. Internal dynamics and delays in adoption within the School of Nursing	3.1. AA as a non-priority agenda item	*“I do not remember it being a formal agenda item… other issues were prioritized.”* (E1)
	3.2. Operational uncertainties overshadowing agreement	*“We did not have a legal team to consult… only other faculty.”* (E1)
	3.3. Narrow conceptualization of retention	*“We basically think about the PED stipend… trying to prioritize that.”* (E1)
4. Ethical dilemmas, misconceptions, and conceptual limits in AA construction	4.1. Confusion between race and socioeconomic disadvantage	*“Poor white students also struggle… how do we maintain fairness?”* (E4)
	4.2. Fear of institutional repercussions	*“We worried about the impact on indicators… it was a real concern.”* (E2)
	4.3. Resistance framed as caution or neutrality	*“We needed to be careful… to avoid harming anyone.”* (E4)

## Discussion

The findings of this study highlight the complexity of implementing AA policies in a graduate nursing program embedded within a broader institutional context marked by racial inequities, administrative ambiguities, and uneven organizational readiness. Rather than functioning as a linear process, the adoption of AA unfolded as a negotiated and, at times, contradictory trajectory shaped by managers’ conceptual understandings, institutional pressures, and structural constraints. This discussion interprets these findings considering the literature on racial equity in higher education, institutional racism, and governance in postgraduate programs.

### Fragmented conceptualizations and their implications

A central contribution of this study lies in demonstrating how divergent understandings of AA shaped managerial decision-making. Some managers framed AA as a form of historical and ethical reparation grounded in racial justice, aligning with scholarship that positions such policies as essential for confronting systemic racism in Brazilian education (12, 13). Others, however, described affirmative action in highly abstract or race-neutral terms, emphasizing notions of general fairness or administrative equalization. This conceptual ambiguity echoes findings from Brazilian and international studies showing that race-neutral interpretations can dilute the reparative intent of AA and reinforce meritocratic narratives that obscure systemic inequities (14, 37).

These divergent framings were not merely semantic differences; they shaped how managers perceived the legitimacy, urgency, and scope of policy implementation. For example, the recurring dilemma regarding whether AA should also benefit socioeconomically disadvantaged White students illustrates how common-sense notions of equity can conflict with the race-specific goals of affirmative action. Similar tensions have been described as “color-evasive” discourses (38), which maintain a façade of neutrality while reproducing racial hierarchies.

### Institutional ambiguity and uneven implementation

Another major finding concerns the gap between central institutional discourse and concrete implementation practices. Although the university expressed support for AA policies, the absence of binding regulations produced substantial variability across units, resulting in delays and inconsistencies. This phenomenon reflects what Ahmed (39) describes as the “non-performativity” of diversity policies: institutions articulate commitments that are not necessarily accompanied by structural mechanisms capable of producing change.

In this study, managers reported feeling both encouraged and unsupported by university leadership, as responsibility for implementation was devolved to individual graduate programs. Exposure to units more advanced in adopting AA created a mixture of discomfort and motivation, highlighting how institutional comparison can both mobilize and constrain efforts to enact equity-oriented change.

### Internal dynamics and organizational inertia

Within the Graduate Nursing Program, the implementation of AA was shaped by competing priorities, operational uncertainties, and the absence of explicit guidance or legal consultation, contributing to delays and organizational inertia. Even when managers expressed ethical agreement with AA, the lack of procedural clarity—particularly regarding the wording of the admissions call, legal safeguards, and implications for evaluation—resulted in cautious or hesitant responses.

This finding aligns with literature showing that organizational change in higher education is often constrained by bureaucratic inertia, fragmented governance structures, and risk-averse cultures (40). The study also highlights that discussions of retention were disproportionately centered on financial mechanisms (e.g., PED stipends), revealing a narrow conceptualization that overlooks psychosocial, pedagogical, and structural dimensions known to affect the success of students from historically marginalized groups (4, 15).

### Contradictions and the role of racial homogeneity among managers

A central contradiction emerging from the analysis concerns the racial homogeneity of the managerial group, composed entirely of White women. This homogeneity is not incidental; it mirrors broader patterns of racial inequality in Brazilian academia, where White faculty disproportionately occupy leadership and decision-making positions (41).

Such positionality likely shaped how managers interpreted AA, framed equity dilemmas, and assessed institutional risks. Research on institutional racism indicates that predominantly White leadership groups may unintentionally reproduce discourses of neutrality, meritocracy, or administrative caution, even when they are broadly supportive of equity-oriented initiatives (42). In this study, concerns about CAPES indicators, time-to-degree, and retention metrics surfaced as salient sources of hesitation, illustrating how structural pressures intersect with racialized academic cultures to shape institutional decision-making. Acknowledging this contradiction does not diminish the contributions of the managers; rather, it offers a critical lens for understanding how whiteness operates institutionally—structuring notions of fairness, shaping comfort with change, and influencing the prioritization of equity agendas within graduate education.

### Implications for policy and practice

This study offers several implications for graduate programs implementing AA policies:

1. *Conceptual clarity is essential*. Without shared understandings of the purpose of AA, implementation becomes inconsistent and vulnerable to race-neutral reinterpretations.
2. *Institutional leadership must provide enforceable guidelines*. Supportive discourse is insufficient without structural mechanisms that ensure alignment across programs.
3. *Retention must be understood as multidimensional*. Financial support is necessary but not sufficient; psychosocial, academic, and structural support mechanisms are equally critical.
4. *Leadership diversity matters*. Increasing the racial diversity of decision-making bodies may strengthen institutional capacity to implement reparative policies with integrity.
5. *Capacity-building is urgent*. Training on racial equity, institutional racism, and affirmative action can help managers navigate conceptual and ethical dilemmas more effectively.

### Limitations

This study has limitations. The sample, although appropriate for qualitative research and supported by theoretical saturation, consisted exclusively of White women managers. While this composition reflects the demographic profile of leadership within the program, it may limit the range of perspectives captured. Additionally, the study focused on a single graduate program within a public university, and institutional dynamics may vary across contexts. Nevertheless, these limitations also illuminate structural inequities in academic governance and the challenges inherent in implementing racial equity policies.

## Conclusion

This study sought to understand the challenges to the effective implementation of AA policies in the Graduate Nursing Program at the School of Nursing, University of Campinas, based on the perspectives of academic managers who served between 2015 and 2024. The analysis revealed that, although there is broad recognition of the importance of AA as a mechanism for advancing racial equity, its implementation was hindered by institutional barriers, conceptual dilemmas, and heterogeneous perceptions that limited its consolidation as a structuring policy. The main obstacles identified included the absence of continuous training, concerns about CAPES evaluation indicators, the lack of binding regulations, insufficient legal support, and the absence of student retention policies.

The implications of this study extend beyond the Unicamp context. The findings demonstrate that the effectiveness of AA depends on their integration into broader institutional projects, anchored in clear and mandatory normative guidelines and accompanied by robust academic and social support strategies. It is recommended that universities advance policies that, in addition to access measures, incorporate strong student retention mechanisms—such as complementary scholarships, tutoring, psychosocial support, and ongoing faculty development in racial equity—as well as the establishment of permanent inclusion committees to ensure coherence, monitoring, and continuity.

By situating the experience of PPG-Enf–Unicamp in dialogue with international efforts to promote equity in health (43, 44), this study expands the global repertoire on the challenges of postgraduate education in contexts of structural inequality, particularly in the Global South. Its originality lies in analyzing the perceptions of academic managers, offering a privileged lens on how institutional disputes, meritocratic conceptions, and normative gaps shape the implementation of AA in nursing—a field still underexplored in the literature.

The consolidation of AA policies in postgraduate education is not merely an administrative goal; it is an indispensable condition for transforming the profile of health professionals and strengthening more equitable, diverse, and socially committed health systems. Addressing racial inequalities in higher education cannot be postponed or treated as a secondary agenda. It is an ethical, political, and scientific imperative. Affirmative action is not a concession but an urgent commitment to social justice and to the future of a truly inclusive health science.

Finally, recognizing that retention policies are essential to the effectiveness of access measures underscores that these dimensions cannot be treated separately. Affirmative action policies must therefore be understood as comprising two inseparable components: access and retention. Ensuring entry into higher education is only the first step; guaranteeing the conditions for students to remain, develop, and successfully complete their academic trajectories is what truly consolidates the commitment to inclusion.

## Data Availability

The raw data supporting the conclusions of this article will be made available by the authors, without undue reservation.
